# Eribulin Activates the cGAS-STING Pathway via the Cytoplasmic Accumulation of Mitochondrial DNA[Fn fn5]

**DOI:** 10.1124/molpharm.121.000297

**Published:** 2021-10

**Authors:** Charles S. Fermaintt, Leila Takahashi-Ruiz, Huiyun Liang, Susan L. Mooberry, April L. Risinger

**Affiliations:** Department of Pharmacology (C.S.F., L.T.-R., H.L., S.L.M., A.L.R.), and Mays Cancer Center (C.S.F., S.L.M., A.L.R.), University of Texas Health Science Center San Antonio, San Antonio, Texas

## Abstract

Microtubule-targeting agents (MTAs), including both microtubule stabilizers and destabilizers are highly effective chemotherapeutic drugs used in the treatment of solid tumors and hematologic malignancies. In addition to the shared ability of all MTAs to block cell cycle progression, growing evidence shows that different agents of this class can also have mechanistically distinct effects on nonmitotic microtubule-dependent cellular processes, including cellular signaling and transport. Herein, we test the biologic hypothesis that MTAs used in the treatment of triple-negative breast cancer (TNBC) can differentially affect innate immune signaling pathways independent of their antimitotic effects. Our data demonstrate that the microtubule destabilizer eribulin, but not the microtubule stabilizer paclitaxel, induces cGAS-STING–dependent expression of interferon-*β* in both myeloid and TNBC cells. Activation of the cGAS-STING pathway by eribulin was further found to be mediated by the accumulation of cytoplasmic mitochondrial DNA. Together, these findings provide mechanistic insight into how eribulin can induce innate immune signaling independent of its antimitotic or cytotoxic effects.

**SIGNIFICANCE STATEMENT:**

Microtubule-targeting agents (MTAs) are often used in the treatment of breast cancer and have been used in combination with immune checkpoint inhibitors to improve efficacy. Although all clinically approved MTAs share an antimitotic mechanism of action, their distinct effects on interphase microtubules can promote differential downstream signaling consequences. This work shows that the microtubule destabilizer eribulin, but not the microtubule stabilizer paclitaxel, activates the cGAS-STING innate immune signaling pathway through the accumulation of mitochondrial DNA in the cytoplasm.

## Graphical abstract

**Figure:**
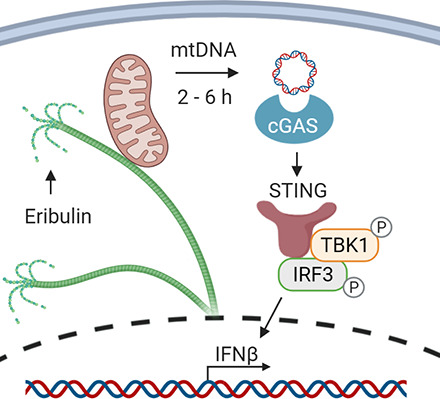

## Introduction

Microtubule-targeting agents (MTAs) are a mainstay in the treatment of many malignancies, including breast cancer (Dumontet and Jordan, 2010). MTAs have been historically classified as microtubule stabilizers or destabilizers based on the biochemical mechanism by which they disrupt microtubule dynamics. Microtubule stabilizers, including the taxanes and epothilones, promote the net polymerization of microtubules, whereas microtubule destabilizers, including the vinca alkaloids and eribulin, promote net microtubule depolymerization (Steinmetz and Prota, 2018). Although MTAs have long been thought to exert their anticancer effects primarily through inhibition of mitosis, growing evidence demonstrates that their nonmitotic effects contribute significantly to their anticancer efficacy (Komlodi-Pasztor et al., 2011, 2012; Field et al., 2014; Kaul et al., 2019b). Mechanistically distinct MTAs can also differ from one another in their nonmitotic effects on oncogenic signaling pathways, and such differences could be important factors in their clinical efficacy (Karbowski et al., 2001; Dybdal-Hargreaves et al., 2017; Kaul et al., 2019b).

In addition to their impact directly on cancer cells, MTAs also have differential effects on both innate and adaptive immune cell populations implicated in antitumor immunity (Fong et al., 2019). Although some of the compound or class-specific immunomodulatory effects of MTAs, such as the ability of paclitaxel to directly activate toll-like receptor 4–dependent signaling (Rajput et al., 2013; Wanderley et al., 2018), have been extensively explored, many of the underlying mechanisms of how MTAs elicit distinct immunologic effects are unknown. A more complete interrogation of the effects of MTAs on immune signaling pathways is warranted given the recent clinical evaluations of MTAs, including taxanes and eribulin, in combination with immune checkpoint inhibitors to enhance the therapeutic response in metastatic triple-negative breast cancer (TNBC) (Mittendorf et al., 2020; Schmid et al., 2020; Tolaney et al., 2021). Collectively, these observations prompted us to test the overall biologic hypothesis that MTAs have different effects on innate immune signaling pathways. Herein, we show that eribulin, but not paclitaxel, induces the cGAS-STING–dependent expression of interferon (IFN) *β* and downstream interferon-stimulated genes (ISGs) in both immune and TNBC cells through the cytoplasmic accumulation of mitochondrial DNA (mtDNA) independent of mitotic arrest or the initiation of apoptosis.

## Materials and Methods

### Cells and Reagents

BT-549 cells were obtained from the Georgetown University Lombardi Comprehensive Cancer Center, Washington, DC. CAL-51 cells were purchased from Creative Bioarray (Westbury, NY). All other cell lines were purchased from the American Type Culture Collection (Manassas, VA). All TNBC lines were authenticated by short tandem repeat (STR) -based profiling (Genetica DNA Laboratories, Cincinnati OH). Primary wild-type bone marrow–derived macrophages (BMDMs) were generated from BALB/c or C57BL/6 mice purchased from Envigo (Indianapolis, IN) as previously described (Hasan et al., 2013). C57BL/6 *Sting* ^gt/gt^ BMDMs were obtained from animals purchased from Jackson Laboratories (Bar Harbor, ME). BMDMs cells were maintained in Dulbecco’s modified Eagle’s medium (Gibco, Grand Island, NY) supplemented with 10% FBS (Corning, Corning, NY) and 50 μg/ml gentamycin (LifeTechnologies, Carlsbad, CA). THP-1, HCC1937, HCC1806, BT-549, MDA-MB-436, and CAL-51 cells were maintained in RPMI 1640 media (Corning) supplemented with 10% FBS and 50 μg/ml gentamycin. HCC1937 cells depleted of mitochondrial DNA (Rho^0^) were generated by culturing cells in 150 ng/ml ethidium bromide (EtBr) for 5 days as described (White et al., 2014). All cell lines were routinely checked for mycoplasma contamination. Drugs, ligands, and inhibitors used in this study include eribulin (Eisai Inc. Woodcliff Lake, NJ), paclitaxel (Sigma Aldrich, St Louis, MO), HT-DNA (Sigma Aldrich), ruxolitinib (Invivogen, San Diego, CA), BX795 (Invivogen), TPCA-1 (Sigma Aldrich), SP600125 (Sigma Aldrich), and H-151 (Tocris, Minneapolis, MN), which were all dissolved in DMSO (Fisher, Hampton, NH) and stored at −20°C.

### siRNA Transfection, RNA Extraction, and qRT-PCR

Treatment with immune pathway inhibitors was initiated 4 hours before treatment with eribulin or HT-DNA followed by continuous treatment with the inhibitor for the indicated time. For *IFNAR* knockdown experiments, THP-1 cells were transfected for 48 hours with siRNAs (Sigma Aldrich) against *IFNAR1* (SASI_Hs01_00121376, SASI_Hs02_00302973) and *IFNAR2* (SASI_Hs01_00208506, SASI_Hs01_00208505) and compared with scramble control (SIC002) using the RNAiMAX reagent (Invitrogen, Carlsbad, CA). Extraction of RNA from cells was done using TRIzol (Ambion, Austin, TX) as indicated by the manufacturer. cDNA was synthesized with iScript cDNA synthesis kit (Bio-Rad, Hercules, CA) and analyzed using a Bio-Rad CFX qRT -PCR using iTaq Universal SYBR Green Supermix (Bio-Rad). mRNA fold change was calculated using the 2^−ΔΔCt^ method, in which *GAPDH* was used as the control. All treated and stimulated samples were compared with their corresponding vehicle or mock control, which was set to a relative value of 1. All experiments were performed as two technical replicates with a variability cutoff of less than 1 Ct within each of two independent biologic replicates. The range of the biologic replicate values is represented in every bar graph. Data were subject to ordinary one-way ANOVA or two-way ANOVA for statistical analysis as described in figure legends. All primers were ordered from Sigma Aldrich, and sequences can be found in Supplemental Table 1.

### Cell Cycle and Caspase 3/7 Cleavage Flow Cytometry Assays

For cell cycle analysis, treated cells were harvested on ice by collecting the media and scraping the adhered cells before centrifuging at 500*g* for 5 minutes at 4°C to pellet both the adherent and nonadherent cells. Cells were mixed with Krishan’s reagent [50 µg/ml propidium iodide, 1 mg/ml sodium citrate, 0.3% (v/v) IGEPAL CA-630, and 20 µg/ml ribonuclease A] and analyzed using a Guava Muse Cell Analyzer (Luminex, Austin, TX) and expressed as percentage of cells in G_1_, S, and G_2_/M based on their DNA content, where error bars represent the range among biologic duplicates. For caspase 3/7 cleavage, adherent treated cells were incubated at 37°C for 30 minutes in a 50-μl/ml Caspase 3/7 green reagent (Thermo-Fisher, Waltham, MA) solution. Cells were subsequently rinsed with PBS and analyzed using a Guava Muse Cell Analyzer (Luminex) and expressed as percentage of cells alive and apoptotic, where error bars represent the range in measured values in biologic duplicates.

### Interferon-*β* Flow Cytometry Assay

THP-1 or HCC1937 cells were treated with 1 µl/ml GolgiPlug (555029; BD Biosciences, Franklin Lakes, NJ) with 100 nM eribulin or the DMSO vehicle control for 2–6 hours. Cells were then collected, blocked with TruStain FcX block (BioLegend, San Diego, CA), and stained with Zombie NIR viability dye (BioLegend) for 15 minutes in the dark. After fixation and permeabilization, cells were incubated with anti-IFN*β-*fluorescein isothiocyanate(FITC) (PBL Assay Science, Piscataway, NJ) for 20 minutes in the dark. Intracellular IFN*β* staining in live cells was determined using the Cytek Aurora at the University of Texas Health Science Center San Antonio Flow Cytometry Facility and analyzed with FlowJo software (Becton, Dickinson and Company, Ashland, OR).

### Transient Transfection and Immunoblotting

Ectopic expression of RFP -cGAS in CAL-51 cells was achieved by transfecting 2.5 µg of the pTRIP-CMV-RFP-FLAG-cGAS [Addgene (Watertown, MA) # 86676] plasmid using the LipofectAMINE 3000 (Invitrogen) reagent for a minimum of 16 hours. For immunoblotting, cells were lysed in cell extraction buffer (Life Technologies) containing 1× protease inhibitor cocktail (Sigma Aldrich) and 1 mM phenylmethylsulfonyl fluoride (PMSF) (Sigma Aldrich). Equal amounts of total protein were resolved by SDS-PAGE on a 10% gel and transferred onto a polyvinylidene fluoride (PVDF) Immobilon-FL membrane (Millipore, Burlington, MA), and relative protein levels were evaluated by immunoblotting for cGAS (1:500 dilution, D1D3G; Cell Signaling Technology, Danvers, MA), STING (1:500 dilution, D2P2F; Cell Signaling Technology), TBK1 (1:500 dilution, D1B4; Cell Signaling Technology), P-TBK1 (Ser172) (1:500 dilution, D52C2; Cell Signaling Technology), and *β*-tubulin (1:10,000 dilution, T4026; Sigma Aldrich) as primary antibodies and species-specific IRDye as secondary antibodies (1:5000 dilution, LI-COR biosciences, Lincoln, NE). Immunoblots were visualized using a LI-COR Biosciences Odyssey Fc Imager.

### Intracellular Tubulin Polymerization Assay

Assessment of intracellular tubulin polymerization in nonadherent THP-1 cells in response to MTAs was done as described previously (Giannakakou et al., 1997). In short, THP-1 cells were washed with 37°C PBS and lysed at 37°C for 5 minutes in a hypotonic lysis buffer [1 mM MgCl_2_, 2 mM EGTA, 0.5% (v/v) Nonidet P-40 (Shell), 50 mM Tris-HCL pH 6.8, and 1× protease inhibitor cocktail]. The lysate was then centrifuged at 14,400*g* for 10 minutes at 37°C to separate soluble tubulin heterodimers (supernatant) from polymerized microtubules (pellet). The pellet was resuspended in an equal volume to the supernatant, and 10% of each fraction was subjected to SDS-PAGE and immunoblotting using *β*-tubulin (1:2000 dilution, T4026; Sigma Aldrich) as the primary antibody and anti-mouse IRDye as the secondary antibody (1:5000 dilution; LI-COR biosciences). Immunoblots were visualized using a LI-COR Biosciences Odyssey Fc Imager.

### Microtubule Indirect Immunoﬂuorescence and Mitochondrial Staining

BMDM cells grown on coverslips were fixed and permeabilized using cold 100% methanol and immunostained using anti–*β*-tubulin (1:400 dilution, T4026; Sigma Aldrich) as primary and anti-mouse FITC (1:200 dilution, F-3008; Sigma Aldrich) as a secondary antibody. HCC1937 cells were stained with 50 nM Mitotracker Red CMXRos (Thermo-Fisher) in serum-free RPMI 1640 for 30 minutes at 37°C, fixed with 4% paraformaldehyde, and permeabilized with 0.5% (v/v) Triton X-100 prior to immunostaining with anti–*β*-tubulin. Samples were imaged on a Nikon (Tokyo, Japan) Eclipse 80i fluorescence microscope with NIS elements using z-stacks at 1000× total magnification.

### Subcellular Fractionation and Detection of Mitochondrial DNA

Detection of mtDNA from cytoplasmic and organelle-enriched fractions was performed as described previously (Wolf et al., 2016). Briefly, cells were scraped into 600 µl of cold disruption buffer (20 mM HEPES, 10 mM KCl, 1.5 mM MgCl_2_, 1 mM EDTA, and 250 mM sucrose) containing 1× protease inhibitor cocktail (Sigma Aldrich) and placed on ice for 5 minutes. Samples were passed through a 22-gauge syringe 30 times followed by a low spin to remove unbroken cells and subsequently subjected to centrifugation at 10,000*g* for 10 minutes at 4°C. The pellet was designated the organelle-enriched fraction (containing nucleus and mitochondria) and the supernatant the cleared cytoplasmic fraction as confirmed by the relative enrichment of VDAC (1:1000 dilution, 4866; Cell signaling Technologies), lamin B1 (1:1000 dilution, ab16048; Abcam), and GAPDH (1:1000 dilution, D4C6R; Cell signaling Technologies) by immunoblotting. DNA was isolated from each fraction using a QiaAmp kit (Qiagen, Germantown, MD) and evaluated for genomic or mitochondrial DNA by qRT-PCR. Cell number was used for normalization between samples, and equal percentages of cytoplasmic and organelle-enriched fractions were compared for each sample to evaluate relative distribution. The primers used to assess mitochondrial genes were ordered from Sigma Aldrich and are in Supplemental Table 1.

### Data Analysis

Data are presented with individual data points representing biologic replicates, and errors are shown as a range of these values. Sample sizes were determined in advance based on previous measurements of accuracy and precision for each of the methodologies used in the current study. These parameters were defined by utilizing validated and literature-grounded positive controls for each assay conducted. All statistical analysis was performed on biologic replicates, with technical replicates performed as an internal check for the rigor of each biologic replicate. The statistical analyses presented in this manuscript were conducted using GraphPad Prism and described in the respective figure legends. In summary, we used a one-way ANOVA with Dunnett’s post hoc test compared with the control condition if only one experimental variable was being measured or two-way ANOVA with Tukey’s post hoc test to compare among each condition if two experimental variables were being measured. If only two samples were being compared, we performed an unpaired *t* test analysis.

## Results

### Eribulin Stimulates the Expression of IFN*β* Independent of Mitotic Arrest or Cytotoxicity

**Fig. 1.**
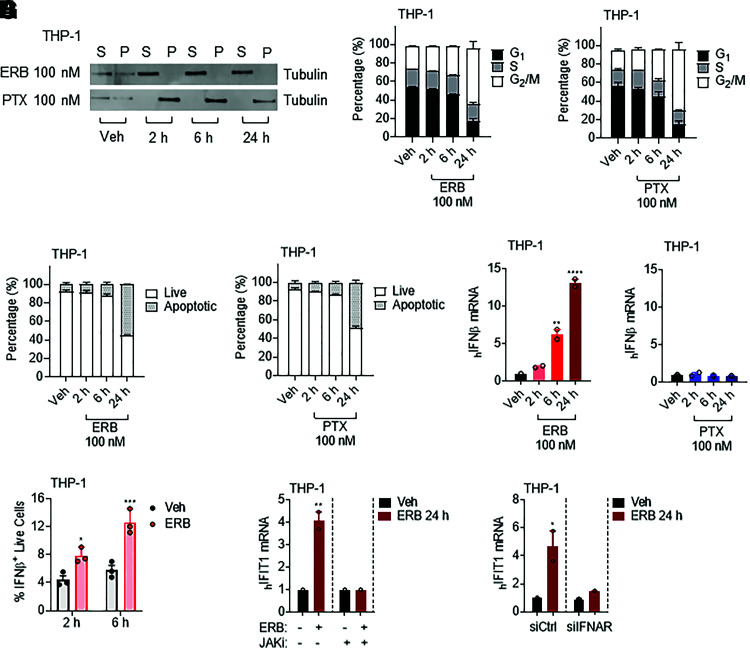
Eribulin but not paclitaxel induces expression of interferon-*β* and interferon-stimulated genes in THP-1 cells. (A) Soluble (S) or polymerized (P) *β*-tubulin in THP-1 cells treated with 100 nM eribulin (ERB) or paclitaxel (PTX) for 2, 6, or 24 hours as compared with DMSO (Veh). (B) Cell cycle analysis of THP-1 cells treated with 100 nM eribulin or paclitaxel for 2, 6, or 24 hours compared with DMSO (Veh) as percentage of cells in G_1_ (black), S (gray), or G_2_/M (white). (C) Caspase 3/7 cleavage in THP-1 cells treated with 100 nM eribulin or paclitaxel for 2, 6, or 24 hours compared with DMSO as percentage of live (white) or apoptotic (gray) cells from two independent experiments with errors denoting range. (D) *IFNβ* mRNA in THP-1 cells treated with 100 nM eribulin or paclitaxel for 2, 6, or 24 hours as compared with DMSO. Significance determined by vehicle compared one-way ANOVA with Dunnett’s post hoc test compared with vehicle. (E) IFN*β* intracellular protein in live cells treated with DMSO or 100 nM eribulin for 2 or 6 hours. Individual data points represent three independent experiments, and significance was determined by two-way ANOVA (time * drug) with Tukeys’s post hoc test. (F and G) *IFIT1* mRNA in THP-1 cells (F) pretreated with 1 µM of ruxolitinib (JAKi) or vehicle for 4 hours and then treated with DMSO or 100 nM eribulin for 24 hours with the inhibitor still present or (G) with siRNA to *IFNAR1/2* (siIFNAR1/2) or a scrambled sequence (siCtrl) for 48 hours followed by treatment with DMSO or 100 nM eribulin for 24 hours. Significance was determined by two-way ANOVA (drug * inhibitor/siRNA) with Tukeys’s post hoc test. qRT-PCR data are shown as individual points from two independent biologic replicates with error bars denoting range. *P < 0.05, **P < 0.01, ****P < 0.0001. Veh, vehicle.

We first determined the time-dependent effects of MTAs on nonadherent THP-1 human monocytic leukemia cells by quantifying the distribution of tubulin in the polymerized microtubule form and soluble tubulin heterodimers by nondenaturing lysis and centrifugation (Giannakakou et al., 1997). When THP-1 cells were treated with 100 nM eribulin or paclitaxel, clinically relevant concentrations of these drugs that promote a rapid microtubule disruption in breast cancer cells (Huizing et al., 1995; Goel et al., 2009; Dybdal-Hargreaves et al., 2017), microtubule depolymerization by eribulin and stabilization by paclitaxel were observed within 2 hours (Fig. 1A). Neither G_2_/M accumulation nor caspase cleavage were observed in MTA-treated cells at either 2 or 6 hours (Fig. 1, B and C). However, 24 hours after either 100 nM eribulin or paclitaxel treatment to THP-1 cells, there was a significant increase in G_2_/M accumulation, and approximately 50% of cells had initiated the process of apoptosis as demonstrated by the activation of caspases 3/7 (Fig. 1, B and C).

At 24 hours, we also evaluated the effects of both drugs on cytokine expression and found that eribulin was distinct from paclitaxel, particularly in its induction of *IFNβ* and ISGs in THP-1 cells, suggesting mechanistic differences between these MTAs that cannot be strictly attributed to their shared antimitotic and apoptotic effects at this time point (Supplemental Fig. 1A). A more detailed time course demonstrated that eribulin indeed promoted the induction of *IFNβ* in THP-1 cells within 6 hours (Fig. 1D), a time when interphase microtubules were disrupted, but prior to mitotic arrest or caspase cleavage. Low expression of *IFNβ* was observed with paclitaxel treatment at each of the time points evaluated with no significant difference from the vehicle control (Fig. 1D). Intracellular flow cytometry further demonstrated an eribulin-mediated increase in IFN*β* protein 6 hours after drug treatment in live cells using a dual gating strategy (Fig. 1E). Together, these data demonstrate that eribulin can induce the expression of IFN*β* at both the mRNA and protein levels in THP-1 cells prior to mitotic accumulation and apoptosis through a mechanism distinct from paclitaxel.

To determine the downstream functionality of eribulin-induced *IFNβ* expression, we further evaluated the concomitant expression of the ISG *IFIT1* over time after the addition of eribulin to THP-1 cells and found that expression also increased significantly 24 hours after treatment (Supplemental Fig. 1B**)**. Further analysis revealed a similar eribulin-mediated induction of another ISG, *OAS1*, with a more modest induction of the ISGs *IFITm3* and *ISG15*, which were not observed in paclitaxel-treated cells (Supplemental Fig. 1C). Although ISG induction coincided with the mitotic arrest and apoptotic effects of eribulin, it is important to note that these shared phenotypes were not sufficient to promote ISG induction in paclitaxel-treated cells. To further test whether the eribulin-mediated induction of ISGs was driven by canonical interferon signaling, which is mediated by JAK-STAT signaling downstream of the binding of interferon to the interferon receptor (IFNAR), we took both genetic and pharmacological approaches (Supplemental Fig. 2A). Indeed, inhibition of JAK kinase activity with ruxolitinib or siRNA-mediated knockdown of IFNAR was sufficient to completely suppress eribulin-mediated expression of the ISG *IFIT1* (Fig. 1, F and G) without inhibiting induction of *IFNβ* expression (Supplemental Fig. 2, B–E). Together, these data demonstrate that either pharmacological or genetic inhibition of the canonical interferon receptor signaling pathway was sufficient to decouple the eribulin-mediated induction of *IFNβ* from downstream ISG induction, further supporting that eribulin induces a functional interferon response.

**Fig. 2.**
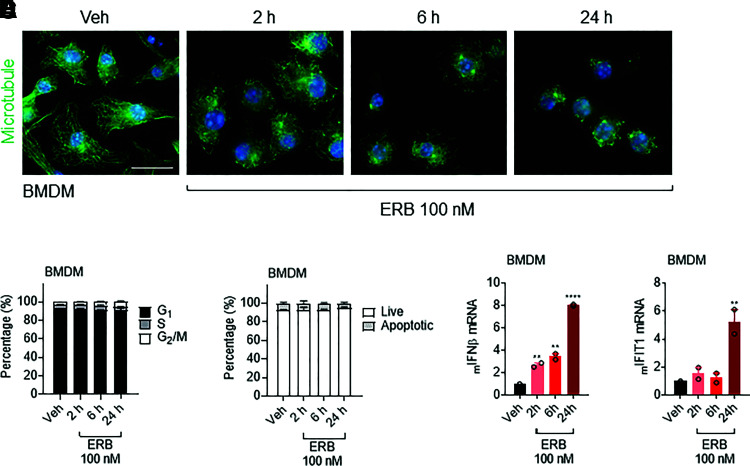
Eribulin-mediated interferon-*β* expression occurs independently of mitotic arrest and caspase cleavage. (A) Immunofluorescence images of microtubules (green) and DNA (blue) in BMDM cells treated with 100 nM eribulin (ERB) for 2, 6, or 24 hours as compared with DMSO (Veh). Scale bar, 10 µm. (B) Cell cycle analysis of BMDM cells treated with 100 nM eribulin for 2, 6, or 24 hours as compared with DMSO as percentage of cells in G_1_ (black), S (gray), or G_2_/M (white) from two independent experiments, with error bars denoting range. (C) Caspase 3/7 cleavage in wild-type BMDM cells treated with 100 nM eribulin for 2, 6, or 24 hours compared with DMSO as percentage of live (white) or apoptotic (gray) cells from two independent experiments with errors denoting range. *IFNβ* (D) and *IFIT1* (E) mRNA in BMDM cells treated with 100 nM ERB for 2, 6, or 24 hours compared with DMSO. qRT-PCR data are shown as individual points from two independent biologic replicates with error bars denoting range. Significance was determined by vehicle-compared one-way ANOVA with Dunnett’s post hoc test. **P < 0.01, ****P < 0.0001. Veh, vehicle.

Previous studies have paradoxically demonstrated a shared upregulation of *IFNβ* in response to either microtubule stabilization or destabilization driven by cytoplasmic nuclear genomic DNA (gDNA) originating from micronuclei formed after prolonged mitotic arrest and slippage (Harding et al., 2017; Mackenzie et al., 2017; Zierhut et al., 2019; Lohard et al., 2020). However, these findings were distinct from the rapid induction of IFN*β* by eribulin, but not paclitaxel, seen in our experiments that occurs prior to mitotic arrest or caspase cleavage. This prompted us to further evaluate the eribulin-mediated IFN response in noncycling primary murine BMDMs. Similar to the results in the THP-1 human cell line, treating murine BMDM with 100 nM eribulin was sufficient to promote depolymerization of the microtubule network within 2 hours (Fig. 2A). However, the primary noncycling BMDMs were refractory to mitotic accumulation or caspase cleavage by eribulin even 24 hours after addition (Fig. 2, B and C). Similar to the THP-1 cells, eribulin-induced *IFNβ* expression was observed within 2 hours in these noncycling cells with subsequent *IFIT1* expression at 24 hours, further demonstrating that eribulin-induced IFN*β* production leads to a functional ISG response independent of mitotic arrest or the initiation of apoptosis (Fig. 2, D and E). Altogether, these data demonstrate that acute eribulin-dependent induction of IFN*β* and downstream ISG signaling in both primary murine and immortalized human immune cells is independent of antimitotic or apoptotic effects, providing a rationale to further explore the mechanistic underpinnings of this induction.

### Eribulin Stimulation of IFN*β* Is Dependent on the cGAS-STING-TBK1 Pathway

**Fig. 3.**
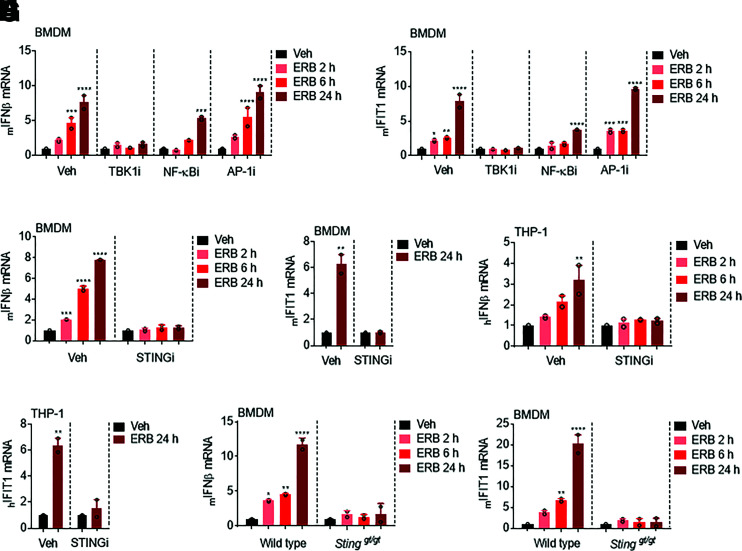
Eribulin-dependent upregulation of interferon-*β* and interferon-stimulated genes is dependent on the STING pathway. *IFNβ* (A) and *IFIT1* (B) mRNA in BMDM cells pretreated with 1 µM of the indicated inhibitor for 4 hours and then treated with 100 nM eribulin (ERB) for 2, 6, or 24 hours as compared with DMSO with the inhibitor still present from two independent experiments with error bars denoting range. *IFNβ* (C and E) and *IFIT1* (D and F) mRNA from BMDM and THP-1 cells pretreated with 1 µM H-151 (STINGi) for 4 hours and then treated with 100 nM eribulin for 2, 6, or 24 hours as compared with DMSO with the inhibitor still present. *IFNβ* (G) and *IFIT1* (H) mRNA in wild-type and *Sting* ^gt/gt^ BMDM cells treated with 100 nM eribulin for 2, 6, or 24 hours. Data are shown as individual points from two independent biologic replicates with error bars denoting range. Significance was determined by two-way ANOVA (time * inhibitor) using a Tukey’s post hoc test. *P < 0.05, **P < 0.01, ***P < 0.001, ****P < 0.0001. Veh, vehicle.

In mammals, the transcription of *IFNβ* is primarily mediated by interferon regulatory factors 3 and 7, NF-*κ*B, and the AP-1 complex (Honda et al., 2006). To preliminarily investigate the likelihood of involvement of each of these transcription factors in the eribulin-mediated expression of *IFNβ*, BMDMs were pretreated with pharmacological inhibitors of each of these pathways prior to eribulin treatment at concentrations that did not promote apoptosis within the timeframe evaluated (Supplemental Fig. 3, A–C). The TBK1 inhibitor BX795 was used to suppress the interferon regulatory factors 3 and 7 signaling pathway, the I*κ*B kinase inhibitor TPCA-1 was used to inhibit NF-*κ*B nuclear localization, and the c-Jun N-terminal kinase inhibitor SP600125 was used to inhibit transcription mediated by the AP-1 complex (Bennett et al., 2001; Podolin et al., 2005; Clark et al., 2009). We found that pretreatment of BMDM with the TBK1 inhibitor completely abrogated eribulin-mediated *IFNβ* and *IFIT1* induction and that this response was also partially inhibited by NF-*κ*B, but not AP-1, inhibition (Fig. 3, A and B).

TBK1 plays a pivotal role in activating the transcriptional expression of *IFNβ* during the process of intracellular DNA sensing by the cGAS-STING pathway (Supplemental Fig. 4A) (Shang et al., 2019). Therefore, we sought to determine whether the cGAS-STING pathway was involved in the eribulin-mediated stimulation of *IFNβ*. To test this, we pretreated BMDM and THP-1 cells with a covalent STING inhibitor, H-151 (Haag et al., 2018), prior to eribulin treatment using a concentration that inhibited TBK1 phosphorylation (Supplemental Fig. 4B) but was not toxic to BMDM or THP-1 cells (Supplemental Fig. 4, C and D). Inhibition of STING signaling by H-151 was sufficient to attenuate the induction of *IFNβ* and *IFIT1* by either eribulin (Fig. 3, C–F**)** or exogenous HT-DNA (Supplemental Fig. 4, E–H) in both primary BMDMs and in THP-1 cells. To further examine the involvement of STING in mediating eribulin-induced expression of *IFNβ*, we generated BMDMs from *Sting ^gt/gt^* mice that contain a missense mutant allele that renders them defective for STING-induced *INFβ* expression (Sauer et al., 2011). Consistent with the results observed with the STING inhibitor, *Sting ^gt/gt^* derived BMDMs failed to upregulate the expression of *IFNβ* and concomitant *IFIT1* when treated with eribulin (Fig. 3, G and H) or exogenous HT-DNA (Supplemental Fig. 4, I and J). Taken together, these studies demonstrate that the eribulin-mediated induction of *IFNβ* and concomitant *IFIT1* expression occurs through a mechanism dependent on the cGAS-STING-TBK1 pathway.

**Fig. 4.**
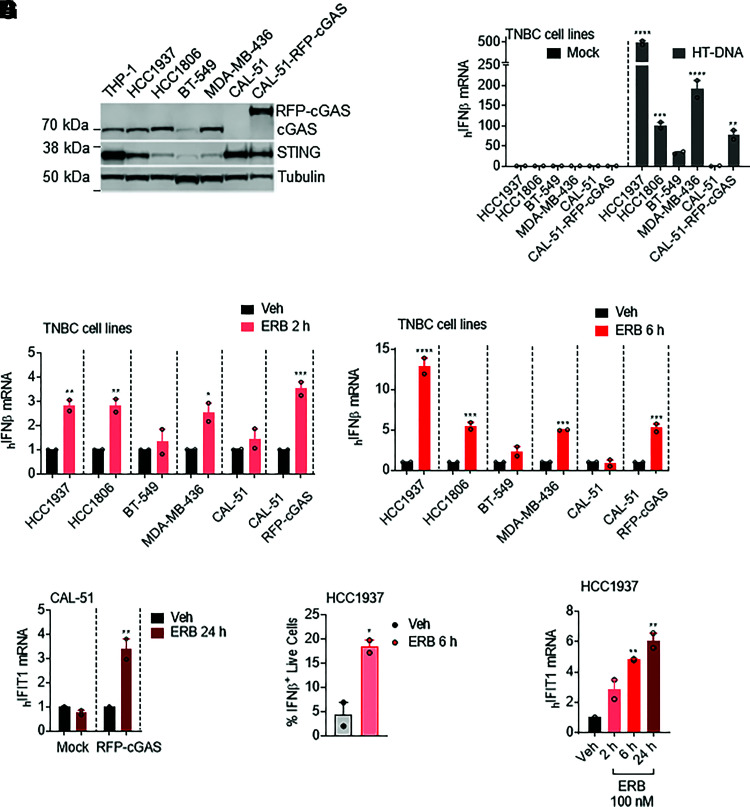
Expression of interferon-*β* by eribulin in TNBC cell lines requires the DNA sensor cGAS. (A) Immunoblot analysis of cGAS, STING, and *β*-tubulin expression in THP-1 and TNBC cells. (B) *IFNβ* mRNA in TNBC cells transfected with 1 µg of HT-DNA for 24 hours or mock-transfected. (C and D) *IFNβ* mRNA in TNBC cells treated with 100 nM eribulin (ERB) for 2 hours (C) or 6 hours (D) as compared with DMSO controls. Significance was determined by two-way ANOVA (cell line * drug) with Tukey’s post hoc test. (E) *IFIT1* mRNA in CAL-51 cells transfected with RFP-cGAS or mock-transfected and treated with DMSO (Veh) or 100 nM ERB for 24 hours. Significance was determined by two-way ANOVA (cGAS * drug) with Tukey’s post hoc test. (F) HCC1937 IFN*β* intracellular protein in live cells treated with DMSO or 100 nM eribulin for 6 hours. Significance determined by an unpaired two-tailed *t* test. (G) Human *IFIT1* mRNA in HCC1937 cells treated with 100 nM eribulin for 2, 6, or 24 hours compared with DMSO. Significance determined by vehicle-compared one-way ANOVA with Dunnett’s post hoc test. Data are shown as individual points from two independent biologic replicates with error bars denoting range. *P < 0.05, **P < 0.01, ***P < 0.001, ****P < 0.0001. Veh, vehicle.

To further interrogate the importance of an intact DNA sensing pathway for eribulin-mediated expression of *IFNβ*, we capitalized on the previous finding that CAL-51 TNBC cells are deficient in cytoplasmic DNA sensing because of a lack of cGAS, which is required for the synthesis of the second messenger cyclic GMP-AMP that functions as a STING agonist (Supplemental Fig. 4A) (Zierhut et al., 2019). Immunoblot analysis of a molecularly diverse panel of TNBC cell lines confirmed that CAL-51 cells express STING but do not express cGAS (Fig. 4A). As expected, each of the cGAS-expressing TNBC cell lines evaluated were capable of inducing *IFNβ* expression when challenged with exogenous HT-DNA, but cGAS-deficient CAL-51 cells were deficient in this response (Fig. 4B). Importantly, ectopic expression of RFP-cGAS (CAL-51-RFP-cGAS) (Fig. 4A) restored the ability of CAL-51 cells to respond to HT-DNA stimulation without introducing gross cytotoxicity (Fig. 4B; Supplemental Fig. 5A). Consistent with the role of the cGAS-STING pathway in the eribulin-mediated induction of *IFNβ* in immune cells, we found that eribulin also promoted the expression of *IFNβ* in cGAS- and STING-expressing TNBC cell lines, as well as the CAL-51 line reconstituted with RFP-cGAS, but was unable to promote *IFNβ* expression in cGAS-deficient CAL-51 cells or low-cGAS-expressing BT-549 cells (Fig. 4, C and D). Consistent with the lack of eribulin-mediated *IFNβ* induction, cGAS-deficient CAL-51 cells also failed to upregulate *IFIT1* when treated with eribulin, although the expression of this ISG was rescued by expression of RFP-cGAS (Fig. 4E). Using flow cytometry, we further demonstrated that live HCC1937 TNBC cells produced IFN*β* protein, which was able to promote downstream *IFIT1* expression within 6 hours of treatment (Fig. 4, F and G). The finding that *IFNβ* expression occurs in these TNBC cells within 6 hours, before mitotic accumulation or cytotoxicity are observed (Supplemental Fig. 5, B–D), further demonstrates the independence of eribulin-mediated interferon expression and downstream ISG expression from its antimitotic mechanism of action. These results demonstrate that eribulin-mediated induction of *IFNβ* and downstream ISGs in immune cells as well as in a molecularly diverse panel of TNBC cells occurs in a cGAS-STING–dependent manner.

### Eribulin Activates the cGAS-STING Pathway by Inducing the Accumulation of Cytoplasmic Mitochondrial DNA

**Fig. 5.**
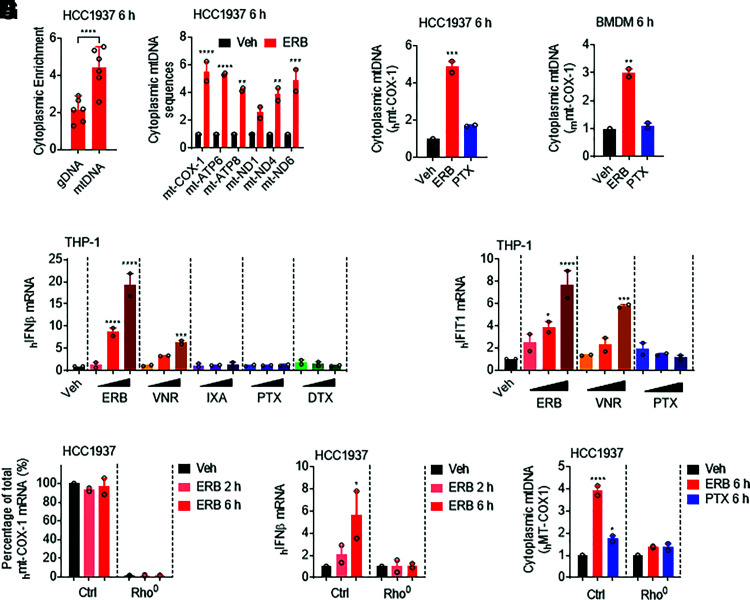
Eribulin promotes cytoplasmic accumulation of mtDNA. (A) qRT-PCR analysis of relative abundance of genomic DNA (gDNA) (*ACTb*, *GAPDH*, *HPRT*, *PGK*, *RPS18*, and *TBP*) and mitochondrial DNA (mtDNA) (*ATP6*, *ATP8*, *COX-1*, *ND1*, *ND4*, and *ND6*) sequences present in the cytoplasm of HCC1937 cells treated with 100 nM eribulin (ERB) for 6 hours. Data are shown as the ratio of gDNA or mtDNA in the cytoplasmic fraction as compared with the organelle-enriched fraction and normalized to the vehicle control as fold change. Significance was determined by one-way ANOVA with Dunnett’s post hoc test. (B and C) COX-1 mtDNA present in the cytoplasm of HCC1937 (B) or BMDM (C) cells treated with 100 nM eribulin or paclitaxel (PTX) for 6 hours as compared with vehicle. Significance determined by vehicle-compared one-way ANOVA with Dunnett’s post hoc test compared with vehicle. (D) *IFNβ* mRNA in THP-1 cells treated with 10, 100, or 1000 nM ERB, vinorelbine (VNR), ixabepilone (IXA), PTX, or docetaxel (DTX) for 24 hours. Significance determined by vehicle-compared two-way ANOVA (drug * concentration) with Tukey’s post hoc test compared with vehicle. (E) *IFIT1* mRNA in THP-1 cells treated with 10, 100, or 1000 nM ERB, VNR, or PTX for 24 hours. Significance determined by vehicle-compared two-way ANOVA (drug * concentration) with Tukey’s post hoc test compared with vehicle. (F) *COX-1* mRNA in control and ethidium bromide cultured (Rho^0^) HCC1937 cells treated with 100 nM eribulin for 2 or 6 hours as compared with DMSO. Significance was determined by two-way ANOVA (Rho status * drug) with Tukey’s post hoc test. (G) *IFNβ* mRNA in control and Rho^0^ HCC1937 cells treated with 100 nM eribulin for 2 or 6 hours as compared with DMSO. Significance was determined by two-way ANOVA (Rho status * drug) with Tukey’s post hoc test. (H) Cytoplasmic *COX-1* DNA present in control and Rho^0^ HCC1937 cells treated with 100 nM eribulin or paclitaxel for 6 hours as compared with DMSO. Significance was determined by two-way ANOVA (Rho status * drug) with Tukey’s post hoc test. Data are shown as individual points from two independent biologic replicates with error bars denoting range. *P < 0.05, **P < 0.01, ***P < 0.001, ****P < 0.0001. Veh, vehicle; ctrl, control.

The dependence of cells on the cytoplasmic cGAS-STING pathway for the induction of IFN*β* in response to eribulin suggests that eribulin could be promoting the accumulation of DNA in the cytoplasm. Since mammalian gDNA and mtDNA serve as the major endogenous cGAS-STING DNA ligands (Härtlova et al., 2015; West et al., 2015), we hypothesized that gDNA and/or mtDNA sequences were enriched in the cytoplasm of cells after treatment with eribulin. To test this hypothesis, we performed differential centrifugation in HCC1937 cells to generate an organelle-enriched (OE) fraction that contained mitochondria and nuclei as evidenced by the enrichment in VDAC and lamin B1, respectively, as well as a cytoplasmic fraction that was enriched for GAPDH (Supplemental Fig. 6A). We then quantified distinct mtDNA and gDNA sequences (six each) that were enriched in the cytoplasmic fraction of HCC1937 cells after a 6-hour treatment with eribulin by qRT-PCR and found a significant increase in the enrichment of mtDNA sequences in the cytoplasm (Fig. 5A). Furthermore, we found that the cytoplasmic enrichment of the *COX*-1 mtDNA sequence specifically occurred in eribulin-treated HCC1937 and BMDM cells as compared with vehicle or paclitaxel treatment (Fig. 5, B and C). These results suggest that the release of mtDNA into the cytoplasmic space could initiate eribulin-mediated induction of the cGAS-STING DNA sensing pathway.

We hypothesized that microtubule destabilization could lead to the release of mitochondrial DNA into the cytoplasm because of the critical role microtubules play in maintaining mitochondrial homeostasis. Intriguingly, microtubule stabilizing and destabilizing agents have previously been shown to elicit differential effects on mitochondrial biogenesis that increase or reduce mitochondrial mass, respectively (Karbowski et al., 2001), and eribulin can effectively disrupt mitochondrial membrane potential within 30 minutes of treatment (Sampson et al., 2016). Therefore, we speculated that eribulin could disrupt mitochondrial homeostasis in a manner that could lead to the cytosolic accumulation of mtDNA. We thus examined the morphology of both microtubules and mitochondria in HCC1937 cells treated for 4 hours with eribulin or paclitaxel. These cells were chosen because of their large size and fine resolution of the microtubule and mitochondrial network. As previously shown for other microtubule destabilizers (Okatsu et al., 2010), we found that eribulin-treated cells acquired a somewhat fragmented mitochondrial phenotype, a feature not observed in paclitaxel-treated cells (Supplemental Fig. 6B). Based on these findings, we hypothesized that other drugs that destabilized microtubules could also promote *IFNβ* and ISG expression. Indeed, when we treated THP-1 cells with increasing concentrations (10–1000 nM) of five different MTAs used in the treatment of breast cancer, we found that only the microtubule destabilizers (eribulin and vinorelbine) but not stabilizers (ixabepilone, paclitaxel, and docetaxel) upregulated *IFNβ* expression (Fig. 5D). Additionally, this *IFNβ* induction by vinorelbine was associated with downstream *IFIT1* expression (Fig. 5E). These data support our hypothesis that destabilization of the interphase microtubule network leads to disruption of mitochondrial homeostasis and subsequent release of mtDNA to drive innate immune activation by these agents.

To directly test the hypothesis of whether the eribulin-mediated release of mtDNA was indeed responsible for the increase in *IFNβ* expression, we took advantage of the fact that EtBr depletes mtDNA while preserving mitochondria themselves (Armand et al., 2004). This method has been used by others to evaluate mtDNA-mediated activation of the cGAS-STING pathway (White et al., 2014; Yamazaki et al., 2020; Hu et al., 2021). We therefore cultured HCC1937 cells in EtBr for 5 days to generate mtDNA-depleted (rho^0^) HCC1937 cells as noted by the depletion of mitochondrial genes in these cells as compared with the untreated controls (Fig. 5F). Strikingly, when mitochondria-deficient HCC1937 rho^0^ cells were treated with eribulin, there was a complete loss of eribulin-mediated *IFNβ* induction (Fig. 5G) that coincided with the absence of eribulin-induced accumulation of mtDNA in the cytoplasm (Fig. 5H). Importantly, HCC1937 rho^0^ cells retained the ability to induce *IFNβ* expression in response to exogenously added HT-DNA (Supplemental Fig. 6C), indicating that these cells retain a functional cGAS-STING pathway. Collectively, these studies demonstrate that cellular mitochondria are critical for mediating the eribulin-dependent accumulation of cytoplasmic mtDNA, which promotes innate immune activation via the cGAS-STING pathway to drive expression of *IFNβ* and ISGs.

## Discussion

MTAs, including the microtubule stabilizer paclitaxel and the microtubule destabilizer eribulin, are some of the most effective agents used in the treatment of metastatic TNBC (Dumontet and Jordan, 2010). Historically, their clinical success has been attributed to their shared ability to suppress mitosis, leading to the apoptosis of rapidly dividing cancer cells. However, accumulating evidence in both patients and preclinical models demonstrates that the anticancer effects of MTAs cannot solely be explained by their shared antimitotic effects and that inhibition of interphase microtubule dynamics significantly contributes to their antitumor properties (Komlodi-Pasztor et al., 2012; Field et al., 2014; Bates and Eastman, 2017). Moreover, different MTAs can elicit distinct effects on cellular oncogenic signaling pathways as well as on mitochondrial homeostasis that may underlie unappreciated clinical effects between these drugs (Karbowski et al., 2001; Dybdal-Hargreaves et al., 2017; Kaul et al., 2019a). Herein, we show that the microtubule destabilizer eribulin induces the cGAS-STING pathway–mediated expression of IFN*β* in innate immune cells as well as in TNBC cells within 2–6 hours by promoting the accumulation of cytoplasmic mtDNA.

One of our most surprising and important findings is that eribulin is distinct from paclitaxel in its ability to promote cytoplasmic accumulation of mtDNA leading to cGAS-STING–dependent interferon and downstream ISG induction. Our results demonstrate that the activation of this immune pathway occurs within 2–6 hours of drug addition and is not dependent on mitotic arrest or the initiation of apoptosis. This is different from studies demonstrating that both microtubule stabilizers and destabilizers can promote activation of the cGAS-STING pathway, specifically during extended periods of mitotic arrest, when genomic DNA is released into the cytoplasm (Mackenzie et al., 2017; Zierhut et al., 2019). Importantly, this previously reported genomic DNA release was dependent on mitosis and not observed in noncycling cells. Our current finding that eribulin rapidly and specifically activates cGAS-STING through mtDNA release in both TNBC and immune cells including those that are terminally differentiated is significant particularly in solid tumors that have a much lower mitotic index than cancer cells in culture (Komlodi-Pasztor et al., 2011).

These findings are timely, as they may contribute to a mechanistic understanding behind the efficacy of combinations of molecularly distinct classes of MTAs, including eribulin, with immune checkpoint inhibition in patients with TNBC (Schmid et al., 2020; Tolaney et al., 2021). The cGAS-STING pathway has been shown to be indispensable for immune checkpoint inhibitors to exert their antitumor effects (Wang et al., 2017), and agents that activate the cGAS-STING pathway have been shown to enhance the efficacy of immune checkpoint therapy in metastatic breast cancer models primarily by priming the immune system to acquire an antitumor phenotype (Chandra et al., 2014; Cheng et al., 2018). These findings have prompted the discovery and development of pharmacological STING agonists; however, our data demonstrate that eribulin and likely a subset of currently approved microtubule destabilizers that innately possess this activity are already in clinical use. Consistent with our findings that the microtubule destabilizer vinorelbine promotes STING activation, preclinical studies have shown an increased response to immune checkpoint inhibitors when combined with vinorelbine (Orecchioni et al., 2018). We speculate that the mitotic-independent activation of the cGAS-STING pathway by eribulin and other microtubule destabilizers could provide an advantage over stabilizers in activating this antitumor-associated immune signaling pathway.

Although our studies demonstrate that the interphase effects of eribulin disrupt mitochondrial localization and promote the release of mtDNA into the cytoplasm and that mtDNA is required for eribulin-mediated activation of the cGAS-STING pathway, the mechanism by which eribulin promotes these mitochondrial effects is implicated from previous studies. The cytoplasmic release of mtDNA under cellular stress can be mediated by the formation of macropores in the mitochondrial outer membrane either through oligomerization of the Bcl-2–associated proteins Bax and Bak (Galluzzi and Vanpouille-Box, 2018) or the formation of VDAC oligomers (Kim et al., 2019). Previous studies have demonstrated that increases in cytoplasmic free tubulin heterodimers, similar to those we observe within 2 hours of eribulin treatment (Figs. 1A and 2A), can directly interact with VDAC channels and disrupt mitochondrial membrane potential (Carre et al., 2002; Maldonado et al., 2010; Rovini, 2019). Additionally, other microtubule destabilizers, including the vinca alkaloids and combretastatin A-4, induce the expression of *NOXA*, which promotes the release and activation of Bax and Bak to form mitochondrial pores in noncycling cells within the same timeframe in which we observe an increase of cytoplasmic mtDNA with eribulin (Bates et al., 2013). Therefore, there is a strong precedent and rationale for how microtubule destabilization can alter mitochondrial permeability through multiple pathways to lead to the innate immune signaling observed in the current study. A rigorous determination of the relative impact of these mechanisms on the release of mtDNA by eribulin will require further investigation.

Together, this work sets the stage for investigation of other innate immune pathways that are specifically modulated by MTAs downstream of their effects on microtubule dynamics and structure. It also specifically prompts interrogation of the adaptive immunologic events downstream of the activation of these innate immune sensing pathways, including whether activation of the cGAS-STING pathway within the tumor microenvironment can serve as a predictive biomarker of response to eribulin, particularly in combination with immunotherapy in TNBC.

## Abbreviations

ACTb: beta actin
AP-1: activator protein 1
Bak: bcl-2 homologous antagonist/killer protein
Bax: bcl-2-associated x protein
BMDM: bone marrow–derived macrophage
Bcl-2: b-cell lymphoma 2
cGAS: cyclic GMP-AMP synthase
COX1: cytochrome c oxidase I
Ctrl: control
EtBr: ethidium bromide
FITC: fluorescein isothiocyanate
GAPDH: glycerol-3-phosphate dehydrogenase
gDNA: genomic DNA
gt: golden ticket
HPRT: hypoxanthine-guanine phosphoribosyltransferase
HT-DNA: herring testes DNA
IFIT: interferon-induced protein with tetratricopeptide repeats
IFN: interferon
IFNAR: interferon receptor
IkB: I kappa B
ISG: interferon-stimulated gene
JAK: Janus kinase
Mt: mitochondrial
MTA: microtubule-targeting agent
mtDNA: mitochondrial DNA
ND: NADH-ubiquinone oxidoreductase chain
NF-*κ*B: nuclear factor *κ*-light-chain-enhancer of activated B cell
NOXA (PMAIP1): phorbol-12-myristate-13-acetate-induced protein 1
OAS1: 2’-5’-Oligoadenylate synthetase 1
PGK: phosphoglycerate kinase 1
PMSF: phenylmethylsulfonyl fluroride
PVDF: polyvinylidene fluoride
qRT-PCR: quantitative real time polymerase chain reaction
RFP: red fluorescent protein
Rho0: cells depleted of mitochondrial DNA
RPS18: ribosomal protein S18
siRNA: small interfering RNA
STAT: signal transducer and activator of transcription
STING: stimulator of interferon genes
STR: short tandem repeat
TBK1: tank binding kinase
TBP: TATA-binding protein
TNBC: triple-negative breast cancer
VDAC: voltage-dependent anion channel
